# Development and Validation of Rapid In-House Diagnostic ELISA Kits for Detection of Human Orthopneumovirus in Clinical Samples

**DOI:** 10.3390/diagnostics12040912

**Published:** 2022-04-06

**Authors:** Ibrahim M. Aziz, Mohamed A. Farrag, Rauf Bhat, Anwar Ahmed, Noorah A. Alkubaisi, Rasha M. Alzayed, Gani Asa Dudin, Fahad N. Almajhdi

**Affiliations:** Department of Botany and Microbiology, College of Science, King Saud University, Riyadh 11451, Saudi Arabia; mfarrag@ksu.edu.sa (M.A.F.); rauf012@yahoo.com (R.B.); anahmed@ksu.edu.sa (A.A.); nalkubaisi@ksu.edu.sa (N.A.A.); rmalzayed@ju.edu.sa (R.M.A.); ganiasa@gmail.com (G.A.D.); majhdi@ksu.edu.sa (F.N.A.)

**Keywords:** human orthopneumovirus, immunization vector, rapid diagnosis, ELISA

## Abstract

Currently, the standard assay employed to diagnose human orthopneumovirus infection is real-time reverse transcriptase PCR assay (rRT-PCR), a costly and time-consuming procedure that requires the manipulation of infectious viruses. In addition to RT-PCR, serological tests can complement the molecular diagnostic methods and have proven to be important tools in sero-surveillance. In this study, we report the development, optimization, and validation of a novel and rapid in-house diagnostic ELISA kit to detect human orthopneumovirus in clinical samples. We developed three sensitive ELISA formats through the immunization of rats with novel recombinant pPOE-F or pPOE-TF vectors. The two vectors expressed either the full-length (pPOE-F) or the truncated form (pPOE-TF) of the fusion (F) protein. The developed ELISA kits were optimized for coating buffer, capture antibody, blocking buffer, sample antigen, detection antibodies, and peroxidase-conjugated antibody, and validated using 75 rRT-PCR-confirmed nasopharyngeal aspirate (NPA) human orthopneumovirus samples and 25 negative samples collected from hospitalized children during different epidemic seasons between 2014 and 2017. Our results indicate that rats immunized with pPOE-F or pPOE-TF showed significant induction of high levels of MPAs. Validation of the ELISA method was compared to the rRT-PCR and the sensitivity hierarchy of these developed ELISA assays was considered from highest to lowest: indirect competitive inhibition ELISA (93.3%) > indirect antigen-capture ELISA (90.6%) > direct antigen-capture ELISA (86.6%). The development of the rapid in-house diagnostic ELISA kits described in this study demonstrates that a specific, rapid and sensitive test for human orthopneumovirus antigens could be successfully applied to samples collected from hospitalized children during different epidemics and can help in the efficient diagnosis of respiratory syncytial viral infections.

## 1. Introduction

Human orthopneumovirus, formerly known as a human respiratory syncytial virus (HRSV), is a prototype member of the genera *Orthopneumovirus*, *Pneumoviridae*, and *Mononegavirales*. It is one of the most important viral pathogens that causes lower respiratory tract infection (LRTI), including bronchiolitis and pneumonia, especially in infants, young children, and immunocompromised elderly patients, causing significant morbidity and mortality globally [1,2,3]. Globally, the virus was estimated to cause approximately 33.1 million infections, with 3.2 million hospital admissions and 59,600 in-hospital deaths in children younger than 5 years of age. The majority of these deaths occurred in developing countries [4]. The healthcare costs due to human orthopneumovirus infections for hospitalized infants were estimated between USD 300 and USD 600 million [5,6,7]. Human orthopneumovirus is divided into two subgroups, A and B, based on antigenic and sequence variations of the attachment glycoprotein (G) and the reactivity with monoclonal antibodies (mAbs) [8,9]. Human orthopneumovirus A and B were circulating together during the epidemic, with a slight predominance of type A. However, a higher prevalence of human orthopneumovirus B was reported periodically [10]. In Saudi Arabia, human orthopneumovirus type A and type B are concurrently circulating, with a slight predominance of human orthopneumovirus type A [11,12].

In comparison with molecular-based approaches, serological-based assays are rapid, accurate, cost-effective, and require less expertise to detect viruses in different clinical samples [13]. The enzyme-linked immunosorbent assay (ELISA) is considered the gold standard of immunoassays and has been particularly useful in rapid and in situ diagnosis. This is attributed to its high sensitivity and specificity, less time consumption, and minimal equipment requirement, and its flexibility to detect and quantify a variety of targets, including antibodies, antigens, proteins, and hormones [14].

Genetic immunization represents a novel approach for vaccination and the generation of polyclonal antibodies (pAbs) and monoclonal antibodies (mAbs) for research purposes [15]. The process of genetic immunization involves the generation of a genetically engineered plasmid containing the gene/antigen(s) against which an immune response is elicited [16,17]. One of the most attractive features of DNA-based immunization is that the desired gene is expressed in vivo, and hence the resultant endogenous antigens elicit T-cell immune responses, namely CD4+ and CD8+ T cells. A strong T helper cell immune response is critical for the induction of high-quality B cells and antibody production [15,18].

In the current study, the production of MPA to F glycoprotein of human orthopneumovirus (Saudi strain) was planned through the immunization of rats with a novel immunization vector (pPOE) expressing either full-length (pPOE-F) or truncated (pPOE-TF) forms of the F protein. Both vectors were generated in our laboratory as described by [19]. The pPOE-F vector expresses the full-length cell-bound F protein that is localized on the host cell surface. The second vector, pPOE-TF, expresses the secreted form of the F protein to induce a potent immune response. Although ELISA kits for human orthopneumovirus are commercially available, in the current study, we addressed detailed protocols of how to generate MPAs and their usage in the optimization and validation of in-house diagnostic kits. These kits will be of high sensitivity to detect local strains of human orthopneumovirus. To achieve our aim, in-house diagnostic ELISA kits (direct and indirect antigen-capture sandwich ELISA and indirect competitive ELISA) were developed and optimized. The validity, specificity sensitivity, reproducibility, and dynamic range of these kits were evaluated using NPA positive human orthopneumovirus samples previously identified using rRT-PCR assay collected from hospitalized children during different epidemic seasons between 2014 and 2017.

## 2. Materials and Methods

### 2.1. Virus, Cells, Media, and Recombinant DNA Plasmids

Human orthopneumovirus type A strain Riyadh 91/2009 (Virology Research Laboratory, College of Science, King Saud University) was propagated in a lung epithelial cell line (A549) (ATCC: CCL-185). The cells were maintained in Dulbecco’s Modified Eagle Medium (DMEM) with 4.5 g/L Glucose, L-glutamine, and Pyruvate (Gibco, Invitrogen, Grand Island, NY, USA), supplemented with 10% fetal bovine serum (FBS) (Gibco, Invitrogen, Grand Island, NY, USA) and 1% penicillin/streptomycin. A virus stock of 10^6^ TCID_50_/_mL_ was prepared for use as a booster for DNA-based immunization. Recombinant vectors, pPOE-F_opt_ and pPOE-TF_opt_, expressing the full-length and secreted F protein form, were used for immunization purposes. Both vectors were enriched and insert verification was attempted using restriction digestion analysis and sequencing, as described by [19].

### 2.2. DNA-Based Immunization and Evaluation of Hyper-Immune Antiserum

Female albino rats (*Rattus norvegicus*) of the Wistar strain, 8–10 weeks old, were obtained and maintained at the animal housing facility of the Zoology Department, College of Science, King Saud University (KSU). The animal experiments were performed following the regulatory guidelines set by the Research Ethics Committee, KSU (4/67/352665). A total of 30 adult female Wistar Albino rats were housed under specific pathogen-free (SPF) conditions for 2 weeks prior to immunization. The rats were then divided into three groups (each in separate cages), each group consisting of 10 rats. Plasmid DNA solutions were diluted in phosphate-buffered saline (PBS) to a final concentration of 100 μg/mL in sterile PBS. DNA-based immunization was performed as previously described by [20], with slight modifications. Two of these groups were inoculated intramuscularly, with 100 µg of pPOE-F or pPOE-TF in sterile PBS, and the third group received pPOE (empty) vector to serve as negative controls. Rats in all groups were immunized three times at 14-day intervals. By day 42, blood was collected from the tail vein of a single rat from each group and the specific antibody response was measured. After a lapse of 3 days from the third immunization (day 42), rats in groups 1 and 2 received a booster dose of 100 µL TCID_50_/_mL_ of human orthopneumovirus Saudi strain with an equal volume of Freund’s incomplete adjuvant by subcutaneous (s.c.) injection. By day 45, all rats were euthanized using chloroform as anesthesia. Blood samples were collected and allowed to coagulate at room temperature. The blood was centrifuged at 3000 rpm for 15 min at 4 °C. The sera were quickly collected, divided into aliquots in pre-labelled Eppendorf tubes. Human orthopneumovirus-F specific antibodies were determined by antigen-capture ELISA using the rat anti-RSV antibody (IgG) ELISA kit, as described in the manufacturer’s instructions (Sino Biological Inc. Beijing, China). ELISA assays were repeated twice with a duplicate. The mean value of the two experiments was calculated along with the standard deviation (SD).

### 2.3. Development of Direct Antigen-Capture ELISA Procedure

Ninety-six-well microtiter plates (Sarstedt, Germany) were sensitized with each MPA from rats immunized with pPOE-F or pPOE-TF and mock rat sera, in duplicate at 1:10 (100 μL/well) in coating buffer (0.05 M carbonate/bicarbonate buffer, pH 9.6). After adsorption at 4 °C overnight, the plates were emptied and 300 μL/well of blocking buffer, 5% skimmed milk protein (SMP) (Difco), was added for 2 h at 37 °C. The blocking solution was discarded, and 50 ng/mL of recombinant human orthopneumovirus F protein (100 μL/well) was diluted with 1% blocking buffer and added to assigned wells. The plates were covered with a plate sealer, mixed gently, and incubated for 1 h at 37 °C. The liquid from each well was aspirated and washed several times with wash buffer (0.05% Tween20 in TBS) and 100 μL/well of HRP-conjugated anti-human orthopneumovirus antibody (1:1000) (Abcam, Cambridge, UK) was added to each well. The plates were then incubated for 30 min at 37 °C, washed, and 3,3′,5,5′-tetramethylbenzidine (TMB) substrate and stop solution were added sequentially; then, the optical density (OD) was measured at a wavelength of 450 nm using an ELX-808 microplate reader (BioTek Laboratories, LL, Shoreline, W.A,). ELISA assays were repeated twice with a duplicate. The mean value of the two experiments along with SD was considered.

### 2.4. Optimization of Various Assay Parameters of Direct Antigen-Capture ELISA

For each MPA from rats immunized with pPOE-F or pPOE-TF tested, the microtiter plate was sensitized with different diluted MPAs and mock rat sera, 100 μL/well of 1:1, 1:2, 1:5, 1:10, 1:20 dilutions in three coating solutions at 4 °C overnight. The coating solutions used were 0.05 M carbonate/bicarbonate buffer (pH 9.6), 0.01 M carbonate/bicarbonate buffer (pH 9.6), and 0.02 M PBS (pH 7.2). Several types of blocking buffers were tested: 1, 3, or 5% casein, 1,3, or 5% bovine serum albumin (BSA), 1, 3, or 5% SMP, and 1, 3, or 5% SMP with 0.01% Tween −20. Three hundred microliters of each blocking buffer were dissolved in PBS and incubated for 2 h with shaking at 37 °C. Five concentrations of recombinant human orthopneumovirus F protein were tested by dilution in 1% blocking buffer, 12.5, 25, 50, 75, or 100 ng/mL (100 μL/well). The optimum dilution of HRP-conjugated anti-human orthopneumovirus antibody used in the ELISA was also determined by using a direct binding assay with recombinant human orthopneumovirus F protein. Different dilutions of HRP-conjugated anti-human orthopneumovirus antibody, 100 μL/well, 1:100, 1:1000, or 1:10,000, were prepared and added to wells coated with 50 ng/mL of recombinant human orthopneumovirus F protein. The ELISA steps were performed as described above.

To determine the suitable incubation time and temperature for the antigen/antibody reaction, each MPA from rats immunized with pPOE-F or pPOE-TF and mock rat sera was sensitized and blocked as described above. Then, 50 ng/mL of recombinant human orthopneumovirus F protein was added for 0.5, 1, or 2 h at 4, 25, or 37 °C. The same incubation periods and temperatures were also tested for the formed complex and HRP-conjugated anti-human orthopneumovirus antibody.

### 2.5. Indirect Antigen-Capture ELISA

A ninety-six-well microtiter plate was coated, the plate was emptied and blocked, recombinant human orthopneumovirus F protein was added and incubated, and the wells were washed as described above. One hundred microliters per well of the dilution 1:1000 of the detecting anti-human orthopneumovirus F antibody, rabbit monoclonal antibody (mAb) (Sino Biological Inc, Beijing, China) in 1% blocking buffer was added to assigned wells. After a 1 h incubation at 37 °C, the wells were washed and 100 μL/well of the dilution 1:10,000 of HRP-conjugated goat anti-rabbit IgG-Fc secondary antibody (Sino Biological Inc. Beijing, China) in 1% blocking buffer was added and incubated for 30 min at 37 °C. The later steps of ELISA were performed as described earlier.

### 2.6. Determination of Various Assay Parameters of Indirect Antigen-Capture ELISA

To determine the optimal dilution of detecting anti-human orthopneumovirus F antibody for each MPA from rats immunized with pPOE-F or pPOE-TF, they were tested by adding 100 μL/well of 1:100, 1:1000, or 1:10,000 dilutions of the detecting anti-human orthopneumovirus F antibody, rabbit mAb in 1% blocking buffer to assigned wells. The wells were washed and 100 μL/well of the dilutions 1:1000, 1:10,000, or 1:20,000 of HRP-conjugated goat anti-rabbit IgG-Fc secondary antibody in 1% blocking buffer was added to the wells. The plate was then processed as described earlier.

### 2.7. Indirect Competitive Inhibition ELISA

ELISA plates were coated with recombinant human orthopneumovirus F protein (100 μL/well, 50 ng/mL) in 0.05 M carbonate/bicarbonate buffer (pH 9.6) and incubated overnight at 4 °C. The coating buffer was discarded, and 300 μL/well of blocking buffer (5% SMP) was added for 2 h at 37 °C. During this step, on a separate plate, the antigen–antibody mixture was prepared by adding 50 μL of recombinant human orthopneumovirus F protein to 50 μL of 1:10 dilutions of MPAs or mock rat sera to assigned wells. The plates were incubated at 37 °C for 2 h. The blocking solution was discarded; 100 μL of the antigen–antibody mixture was added to assigned wells. After a 1 h incubation at 37 °C, the wells were washed 7 times, and 100 μL/well of 1:10,000 dilutions of HRP-conjugated goat anti-rat secondary antibody (Sino Biological Inc. Beijing, China) in 1% blocking buffer was added to each well and the ELISA was performed as described above.

### 2.8. Optimization of Capture-Antigen Concentration and Antigen–Antibody Mixture Concentration

For each MPA from rats immunized with pPOE-F or pPOE-TF, five concentrations of recombinant human orthopneumovirus F protein (100 μL/well, 12.5, 25, 50, 75, 100 ng/mL) were coated; the plate was then processed as described earlier. To determine the optimal dilution of MPAs raised against pPOE-F or pPOE-TF within the antigen–antibody mixture, five dilutions of each MPA were prepared by adding 50 μL of 1:1, 1:2, 1:5, 1:10, or 1:20 to 50 μL/well, 25 ng/mL in 1% blocking to the assigned wells. The ELISA procedures were performed as described above.

### 2.9. Validating the Performance of the Developed ELISA Kits on Clinical Samples

A total of 100 NPA, 75 positives human orthopneumovirus and 25 negatives human orthopneumovirus samples previously identified using rRT-PCR assay, were collected from hospitalized children suffering from acute LRTI at King Khalid University Hospital (KKUH) during different epidemic seasons throughout 2014–2017. These samples were utilized for validation of the developed ELISA (direct and indirect antigen-capture sandwich ELISA and indirect competitive ELISA) as described above. Each sample was tested in triplicate in the same plates to evaluate the intra-assay reproducibility and in different plates to evaluate the inter-assay reproducibility. The specificity and sensitivity of the ELISA kits were assessed by comparing the ELISA results with the results of the same samples in the standard assay, RT-PCR. The specificity and sensitivity were calculated according to the following formulas:

Specificity = True positive/(True positive + False negative)

Sensitivity = True negative/(True negative + False positive)

### 2.10. Statistical Analysis

Statistical Package for Social Science (SPSS) version 22 (SPSS Inc. Chicago, IL, USA) was used for data entry and analysis. Continuous data were presented as mean ± SD for normal variables. An independent *t*-test was used to compare mean differences. A *p* value less than 0.05 was considered to be significant.

## 3. Results

### 3.1. pPOE Vectors Elicited a Significant Antibody Response in Immunized Rats

**Figure 1:**
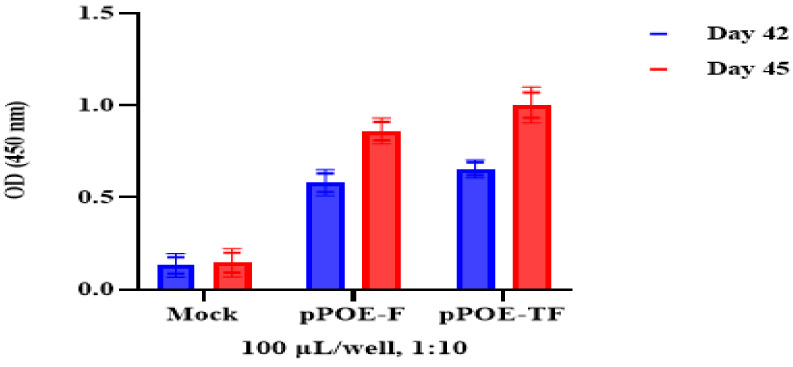
The evaluation of human orthopneumovirus F MPA levels in serum at day 42 and day 45. OD values are the mean of two independent experiments and bars represent means ± SD measured in each group. Differences between rats immunized with pPOE-F and pPOE-TF were statistically analyzed.

To evaluate the levels of anti-human orthopneumovirus F specific antibody using antigen-capture ELISA, serum samples were collected from immunized rats at day 42 and 3 days after the booster immunization dose (day 45). As shown in Figure 1, the antibody detection ELISA revealed the absence of anti-F-specific antibodies in the mock-immunized rats at day 42 (i.e., no background or cross-reactivity of antibodies). Both pPOE-F and pPOE-TF elicited reasonable antibody levels at day 42, with mean OD values of 0.53 ± 0.078 and 0.62 ± 0.098, respectively. At day 45, the rats immunized with pPOE-F or pPOE-TF showed significant induction of high levels of MPAs, with mean OD values of 0.93 ± 0.032 and 0.81 ± 0.031, respectively. There was no significant difference in antibody titers between rats immunized with pPOE-F and pPOE-TF.

### 3.2. Optimization of Various Assay Parameters of Direct Antigen-Capture ELISA

#### 3.2.1. The Coating Buffer

**Figure 2:**
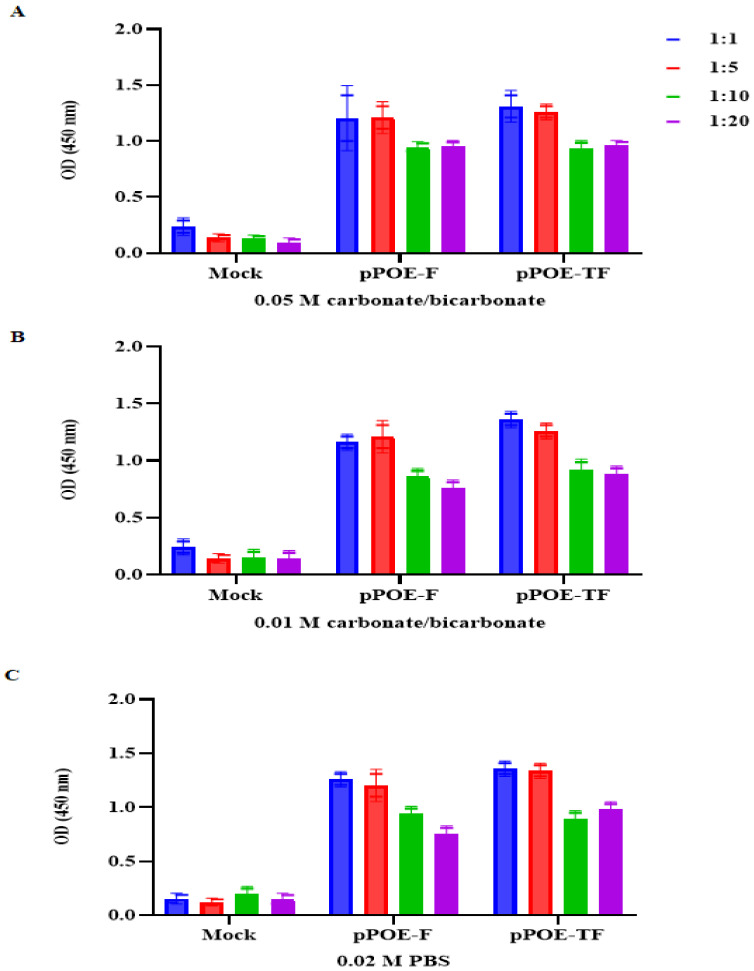
Graph showing optimum dilutions of each MPA in (**A**) 0.05 M carbonate/bicarbonate buffer, (**B**) 0.01 M carbonate/bicarbonate buffer, and (**C**) 0.02 M PBS coating buffers; various dilutions of each MPA were adsorbed in microtiter wells and tested for their ability to bind 50 ng/mL of recombinant human orthopneumovirus F protein. OD values are the mean of two independent experiments and bars represent means ± SD measured in each MPA. Differences between rats immunized with pPOE-F and pPOE-TF were statistically analyzed.

Optimum antisera dilutions were determined by checkerboard titrations and the results were interpreted graphically. To evaluate the binding affinity of MPA to the F protein and the optimal coating buffer, serial dilutions of each capture MPA in three coating solutions, 0.05 M carbonate/bicarbonate buffer, 0.01 M carbonate/bicarbonate buffer, and 0.02 M PBS, were evaluated individually. As depicted in Figure 2A, of the three coating solutions, at 0.05 M carbonate/bicarbonate buffer, the OD values of the 1:1 or 1:5 dilution of capture MPA from rats immunized with pPOE-F or pPOE-TF were high, with an unacceptable background. Meanwhile, the OD values of the dilution 1:10 were high with an acceptable background, with mean OD values of 0.94 ± 0.12 (pPOE-F) and 0.93 ± 0.21 (pPOE-TF). Similarly, at the dilution 1:20 of MPAs from rats immunized with pPOE-F or pPOE-TF, the OD values were 0.95 ± 0.18 and 0.96 ± 0.21, respectively. Decreasing the concentration of carbonate/bicarbonate buffer to 0.01 M and 0.02 M PBS gave comparable results since OD values were similar and with unacceptable background in the ELISA (Figure 2B,C). However, the OD values of the concentration 0.05 M were better than those of the 0.01 M and 0.02 M. Therefore, the dilution 1:10 of MPAs and 0.05 M carbonate/bicarbonate coating buffer were chosen as the optimum working dilutions for further processes in the development of the ELISA kits.

#### 3.2.2. The Blocking Buffer

**Figure 3:**
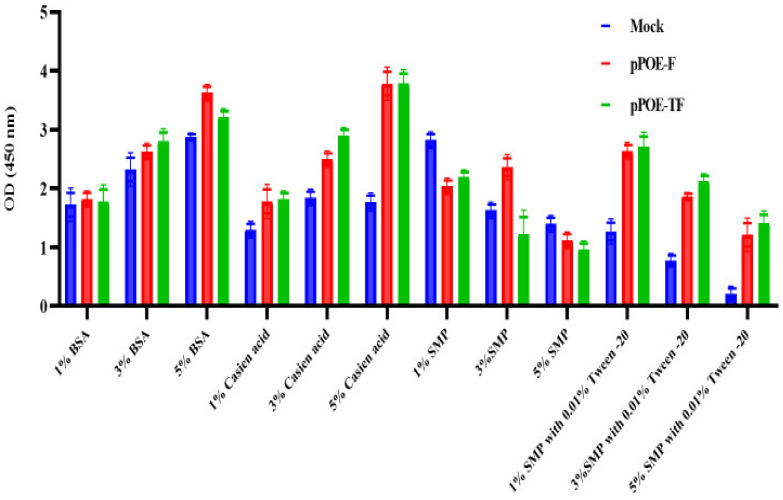
Graph showing optimum blocking buffer. The optimal dilution of each MPA was coated and blocked with different blocking buffers. Recombinant human orthopneumovirus F protein was added and detected by HRP-conjugated anti-RSV antibody in 1% blocking buffer. OD values are the mean of two independent experiments and bars represent means ± SD measured in each MPA. Differences between rats immunized with pPOE-F and pPOE-TF were statistically analyzed.

To optimize the best types of blocking buffer, different blocking buffers were tested, including casein (1, 3, or 5%), BSA (1, 3, or 5%), SMP (1, 3, or 5%), and SMP (1, 3, or 5%)/0.01% Tween −20. As shown in Figure 3, at 1 or 3% of casein and BSA blocking buffers, there is a plateau curve with OD values above 1.5, which is in disagreement with ELISA. Moreover, the OD values of the background were increased upon increasing the concentration of casein to 5%. Consequently, casein and BSA blocking buffers were excluded from further downstream optimization experiments. In a similar fashion, the concentrations of 1 or 3% of SMP and SMP/0.01% Tween −20 yielded a high ELISA signal above 1.5 and an unacceptable background for both MPAs. In contrast, 5% SMP with or without 0.01% Tween −20 yielded a high ELISA signal and an acceptable background for both MPAs. Therefore, the concentration of 5% SMP with 0.01% Tween −20 was chosen as the optimum blocking buffer and working dilution for both MPAs.

#### 3.2.3. The Optimum Concentration of Antigen and HRP-Conjugated Antibody

**Figure 4:**
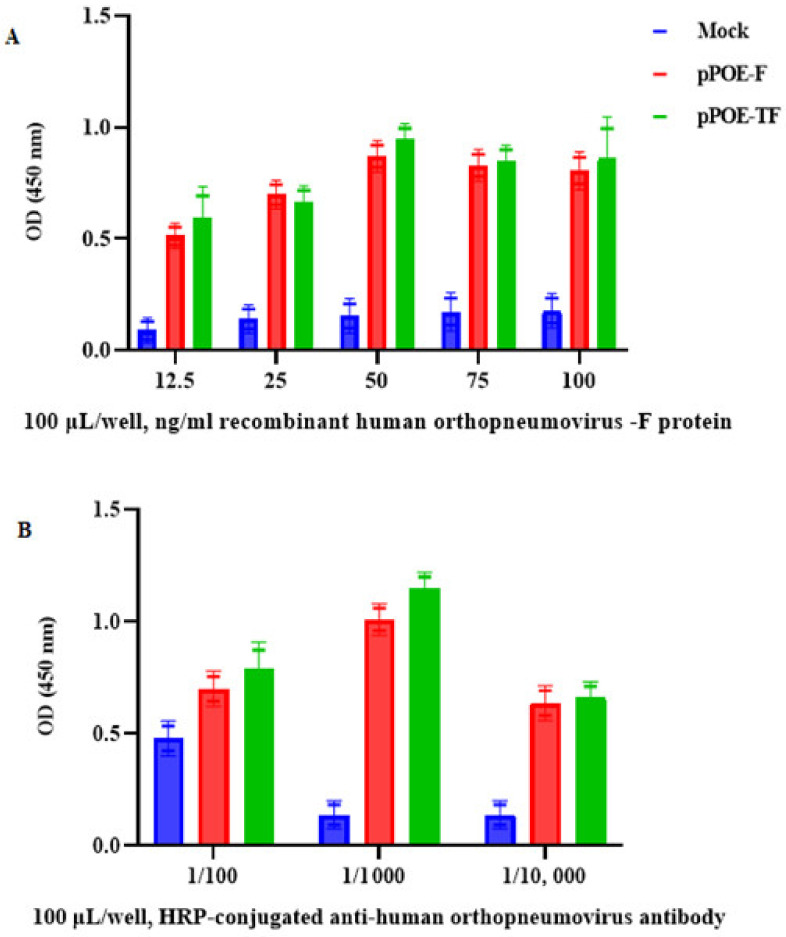
(**A**) Graph showing optimum concentrations of recombinant human orthopneumovirus F protein. Different concentrations of recombinant human orthopneumovirus F protein were added and detected by various dilutions of HRP-conjugated anti-RSV antibody. (**B**) Graph showing the various dilutions of HRP-conjugated anti-human orthopneumovirus antibody. OD values are the mean of two independent experiments and bars represent means ± SD measured in each MPA. Differences between rats immunized with pPOE-F and pPOE-TF were statistically analyzed.

To determine the best dilutions of recombinant human orthopneumovirus F protein, five concentrations of the F protein were prepared (100 μL/well:12.5, 25, 50, 75, and 100 ng/mL). As depicted in Figure 4A, the OD values of F protein were reasonable even at a low concentration (12.5 and 25 ng/mL). Meanwhile, the OD values of MPAs at concentrations of 50 ng/mL were high from rats immunized with pPOE-F (0.87 ± 0.23) or pPOE-TF (0.94 ± 0.12) with an acceptable background as compared with other concentrations. Therefore, it was chosen as the optimum working dilution for both MPAs for both vectors.

To evaluate the optimum dilutions of conjugated antibody, dilutions of 1:100, 1:1000, and 1:10,000 were tested. At dilutions of 1:1000, the OD values of antibody from rats immunized with pPOE-F or pPOE-TF were higher, with mean OD values of 0.96 ± 0.15 and 1.14 ± 0.21, respectively, and an acceptable background as compared with dilutions of 1:100 or 1:10,000 for both antisera (Figure 4B). Therefore, the conjugate dilution of 1:1000 was selected for both antisera for further optimizations.

#### 3.2.4. Incubation Periods and Temperature

**Figure 5:**
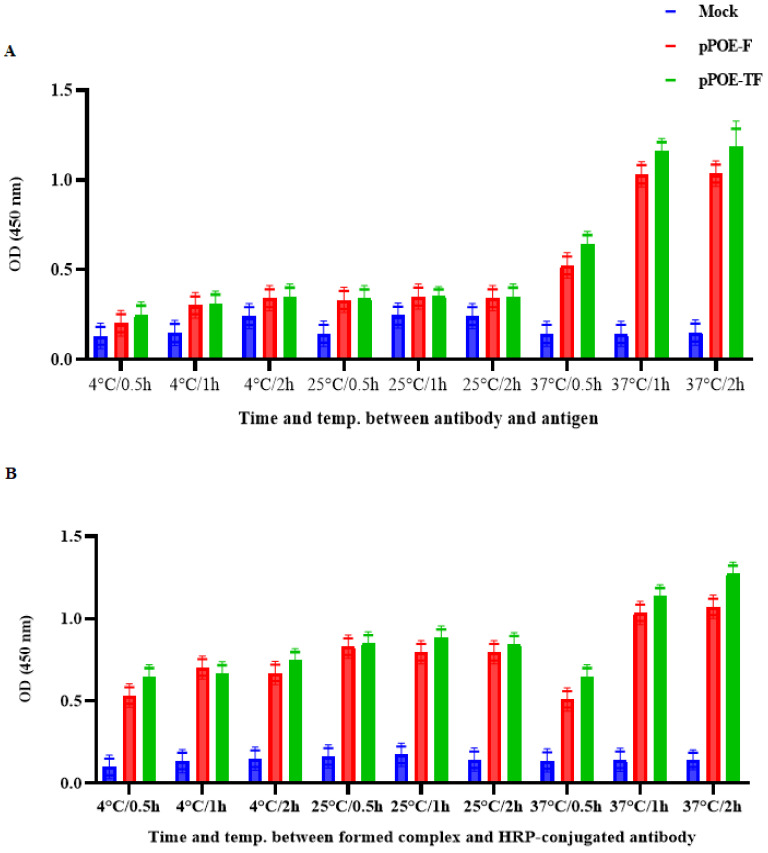
(**A**) Graph showing the suitable time and temperature for the reaction between antibody and antigen and (**B**) between the formed complex and enzymatic conjugate ELISA. The incubation periods and temperature were 0.5, 1, or 2 h at 4, 25, or 37 °C. OD values are the mean of two independent experiments and bars represent means ± SD measured in each MPA. Differences between rats immunized with pPOE-F and pPOE-TF were statistically analyzed.

To optimize the suitable time and temperature for the antigen/antibody reaction and between the formed complex and the conjugate, three incubation periods (0.5, 1, or 2 h) and three temperatures (4, 25, and 37 °C) were compared (Figure 5A,B). The sensitivity of the ELISA for antibodies from rats immunized with pPOE-F or pPOE-TF at 37 °C was higher than that at 4 and 20 °C. At 37 °C, there was a twofold increase in sensitivity between 0.5 and 2 h. At 4 and 25 °C, there was a slight increase between 0.5 and 2 h incubations. Overall, incubation conditions of 1 h at 37 °C yielded a high ELISA signal and acceptable background for both antibodies from rats immunized with pPOE-F or pPOE-TF, with mean OD values of 1.08 ± 0.13 and 1.16 ± 0.11, respectively. Meanwhile, assays of 1 h at 37 °C for the reaction of formed complex and enzymatic conjugate yielded a high ELISA signal and acceptable background for both antibodies from rats immunized with pPOE-F or pPOE-TF, with mean OD values of 0.96 ± 0.22 and 1.09 ± 0.17, respectively. Therefore, incubation conditions of 2 h at 37 °C for the reaction between antibody and antigen and 1 h at 37 °C for the reaction of formed complex and the conjugate were chosen to achieve high sensitivity and optimum convenience. No significant differences in the assay performance were observed between each MPA from rats immunized with pPOE-F or pPOE-TF in various assay parameters utilized for the direct antigen-capture ELISA procedure.

### 3.3. Optimization of Various Assay Parameters of Indirect Antigen-Capture ELISA

**Figure 6:**
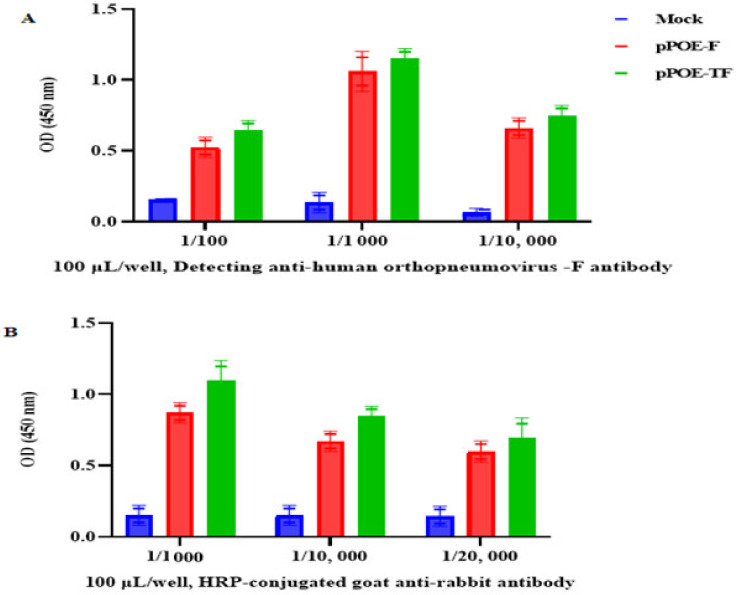
(**A**) Graph showing optimal dilution of detecting anti-human orthopneumovirus F antibody for each MPA; various dilutions for detecting anti-human orthopneumovirus F antibody were adsorbed in microtiter wells and tested for their ability to bind 50 ng/mL of recombinant human orthopneumovirus F protein, and detected by (**B**) various dilutions of HRP-conjugated goat anti-rabbit IgG-Fc secondary antibody in 1% blocking buffer. OD values are the mean of two independent experiments and bars represent means ± SD measured in each MPA. Differences between rats immunized with pPOE-F and pPOE-TF were statistically analyzed.

For the indirect antigen-capture ELISA procedure, the optimal dilution for detecting anti-human orthopneumovirus F antibody for each MPA was determined by using a direct binding assay with the F protein-coated wells. As shown in Figure 6A, we observed an increase in signals from both MPAs from rats immunized with pPOE-F and pPOE-TF, with mean OD values of 1.06 ± 0.09 and 1.12 ± 0.15, respectively. An acceptable background was obtained at the dilution of 1:1000 as compared to 1:100 or 1:10,000 of the detecting anti-human orthopneumovirus F antibody. Similarly, the dilution of 1:1000 of HRP-conjugated goat anti-rabbit IgG-Fc secondary antibody gave the highest OD values and acceptable background for both MPAs from rats immunized with pPOE-F and pPOE-TF, with OD values of 0.87 ± 0.21 and 0.97 ± 0.13. This dilution was adopted as the optimum working dilution for both antisera (Figure 6B).

### 3.4. Optimization of Indirect Competitive Inhibition ELISA

**Figure 7:**
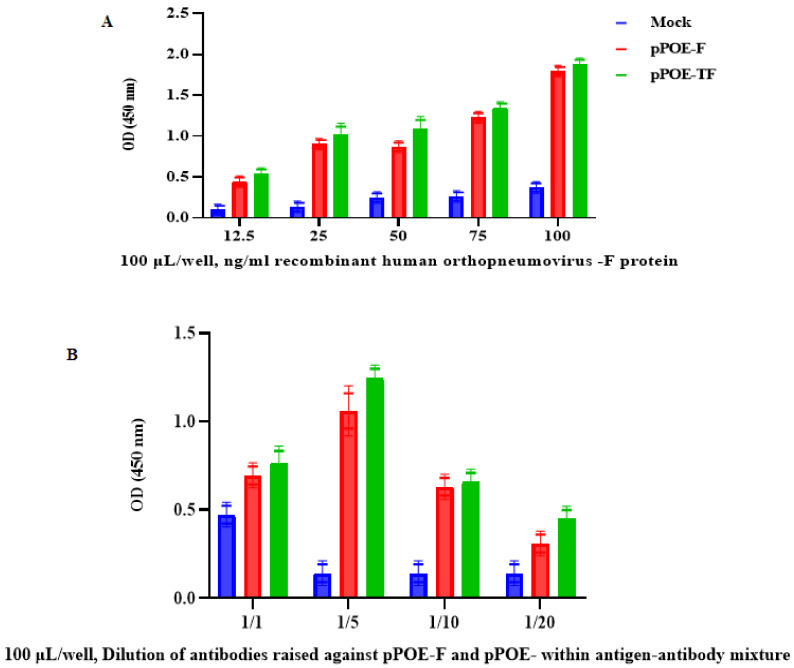
(**A**) Graph showing optimum concentrations of capture recombinant human orthopneumovirus F protein for each MPA; various concentrations of recombinant human orthopneumovirus F protein were adsorbed in microtiter wells and tested for their ability to bind with pPOE-F and pPOE-TF antibodies, and detected using HRP-conjugated goat anti-rat secondary antibody. (**B**) Graph showing optimum dilution of antibodies raised against pPOE-F and pPOE-TF within the antigen–antibody mixture; five dilutions of each MPA were tested by adding 50 μL of 1:1,1:2, 1:5, 1:10, or 1:20 to 50 μL/well, 25 ng/mL in 1% blocking. OD values are the mean of two independent experiments and bars represent means ± SD measured in each MPA. Differences between rats immunized with pPOE-F and pPOE-TF were statistically analyzed.

To optimize the concentration of capture-recombinant human orthopneumovirus F protein for each MPA, five concentrations of recombinant human orthopneumovirus F protein (100 μL/well, 12.5, 25, 50, 75, 100 ng/mL) were coated in coating buffer and tested for their ability to bind with MPAs from rats immunized with pPOE-F or pPOE-TF, and detected by using HRP-conjugated goat anti-rat secondary antibody. As shown in Figure 7A, the sensitivity of the ELISA increased in both MPAs from rats immunized with pPOE-F or pPOE-TF, with OD values of 0.91 ± 0.19 and 1.11 ± 0.13, respectively. An acceptable background was obtained at the concentration of 25 ng/mL as compared to 12.5 ng/mL of recombinant human orthopneumovirus F protein. We found that at 50 ng/mL of the F protein, the OD values did not differ from those obtained with 25 ng/mL of the F protein. At 75 ng/mL of the F protein, the background was unacceptable and the background OD values increased when increasing the F protein concentration to 100 ng/mL. Therefore, the F protein concentration of 25 ng/mL was selected for both antisera. Considering the optimal dilution of MPAs within the antigen–antibody mixture, five dilutions of each MPA from immunized rats were prepared by adding 50 μL of 1:1, 1:2, 1:5, 1:10, or 1:20 to 50 μL/well, 25 ng/mL in 1% blocking. At the dilution of 1:5, the OD values of both MPAs were high and had an acceptable background: 1.11 ± 0.13 (pPOE-F) and 1.24 ± 0.23 (pPOE-TF). The OD values were decreased with an increasing dilution of MPAs to 1:20 of MPAs. Therefore, the dilution of 1:5 was chosen as the optimum working dilution for both MPAs. There were no significant differences between MPAs from rats immunized within various assay parameters of the indirect competitive inhibition ELISA (Figure 7B).

### 3.5. Validating the Performance of the Developed ELISA Kits Using Clinical Samples

To validate the efficiency of in-house diagnostic ELISA kits, the kits were used to detect human orthopneumovirus in positive samples. Due to the secreted nature of the F protein, it elicited a good antibody titer and thus was selected for subsequent experimental validation. The results of the developed ELISA kits were compared with rRT-PCR assay results. For this purpose, a total of 100 NPA samples (75 positive and 25 negatives), previously identified using rRT-PCR assay, were collected from hospitalized children suffering from acute LRTI at KKUH during different epidemic seasons throughout 2014–2017. These samples were processed using the three in-house diagnostic ELISA kits and compared to the rRT-PCR assay. The sensitivity and specificity were also calculated with respect to rRT-PCR. As shown in Table 1, out of the total 100 samples tested, 25 rRT-PCR negative samples were used as the negative standard, and there were 75 rRT-PCR positive samples. Out of the 75 rRT-PCR positive samples 10 samples were found to be negative in the direct antigen-capture ELISA; out of the 25 rRT-PCR negative samples, five were found to be positive in the direct antigen-capture ELISA. The direct antigen-capture ELISA yielded good sensitivity (86.6%) and specificity (80.0%), when using the rRT-PCR as a reference. Concerning the validation of the performance of the indirect antigen-capture ELISA, seven samples out of 75 rRT-PCR positive samples were negative by ELISA, four samples out of 25 rRT-PCR negative samples were positive by ELISA, and the sensitivity and specificity were 90.6% and 84.0%, respectively. With respect to the validation of the performance of the indirect competitive inhibition ELISA, five samples out of 75 rRT-PCR positive samples were negative by ELISA, and three samples out of 25 rRT-PCR negative samples had a positive test, and yielded a sensitivity of 93.3% and specificity 88.0%, for the MPAs from rats immunized with pPOE-TF. The sensitivity hierarchy of these developed ELISA assays was considered from highest to lowest as follows: indirect competitive inhibition ELISA (93.3%) > indirect antigen-capture ELISA (90.6%) > direct antigen-capture ELISA (86.6%).

## 4. Discussion

Human orthopneumovirus is the primary cause of LRTI in children under 5 years of age. It accounts for approximately 33 million cases and up to 100,000 deaths annually. Premature infants, young children, and individuals with underlying risk factors are more prone to experience severe human orthopneumovirus infections, with high morbidity and mortality rates [21,22]. Rapid diagnosis of human orthopneumovirus is important for physicians and clinicians to launch an appropriate therapeutic protocol. In hospital settings, rRT-PCR is widely adopted for the diagnosis of human orthopneumovirus. However, the need for experienced staff and the cost of reagents hinder its use in many developed countries and also in rural areas. In this case, ELISA provides a sensitive, specific, and considerably cheaper approach to diagnose human orthopneumovirus in clinical specimens. Human orthopneumovirus infection can be rapidly diagnosed by the detection of viral antigens in NPAs by rRT-PCR and ELISA during the early stages of upper respiratory infection, namely during the first 5 days of the infection [13,23,24]. Human orthopneumovirus F protein, a type 1 viral protein, has long been considered the most promising target for the preparation of diagnostic kits and antiviral therapeutic agents. This is attributed to its conserved nature in both A and B subgroups and its ability to activate both the humoral and cellular immune responses [25,26]. Thus, in the present study, two recombinant vectors, pPOE-F and pPOE-TF, were injected into rats and the raised MPAs were used to develop an in-house ELISA kit.

The development of the new in-house diagnostic ELISA kits required the evaluation of several parameters. Among these, the first parameter to be optimized was the plate-coating conditions for the antigen or the capture antibodies. Carbonate-type buffers are the most commonly used buffers in the ELISA system [27]. Capture antibodies are immobilized on the plate by passive adsorption, usually using coating buffer at the same pH or slightly above the isoelectric point (pI) (the pH at which the net charge on the protein is zero). Most but not all proteins bind tightly to the polystyrene surface of microplates in alkaline conditions. Thus, selecting a coating buffer between pH 7.4 and pH 9.6 can affect the steric structure of protein/antibody/analyte binding and thus affect their immobilization. Three widely used coating buffers are 0.05 M carbonate/bicarbonate pH 9.6, 0.01 M Tris pH 8.5, and 0.01 M PBS pH 7.2 [28]. In the current study, the coating buffer 0.05 M carbonate/bicarbonate buffer, at pH 9.6, gave the highest absorbance value at A450 and an acceptable background with 1:10 dilutions of rats’ MPAs.

The blocking step is as important as the coating step, which prevents non-specific interactions and stabilizes the biomolecules bound to the well surface of the ELISA plates. It is therefore used to improve the sensitivity of the ELISA by reducing the background and improving the signal-to-noise ratio [29,30]. The two major classes of blocking reagents are proteins (e.g., BSA, SMP, and casein) and detergents (typically non-ionic, such as Tween 20 and Triton X-100) [31]. In the current study, several types of blocking buffers, including 1, 3, or 5% casein, 1, 3, or 5% BSA, 1, 3, or 5% SMP, and 1, 3, or 5% SMP with 0.01% Tween −20, were optimized. The use of 5% casein and BSA blocking buffers leads to high absorbance values above 1.5, which disagrees with ELISA standards. This finding is consistent with a previous study that suggested that some protein blockers, such as casein, can react with antibodies, resulting in a high background. Similarly, some BSA preparations may contain phosphotyrosine and give a high background when using anti-phosphotyrosine antibodies. Moreover, BSA is not compatible with lectin probes as it contains carbohydrates that can increase the non-specific background [31]. In our study, 5% SMP and 5% SMP with 0.01% Tween −20 yielded a high ELISA signal and an acceptable background. In addition, 5% SMP with 0.01% Tween −20 yielded the highest ELISA absorbance with the lowest background compared to the 5% SMP buffer. The same finding was reported previously, where a combination of 5% SMP and 0.1% Tween −20 significantly improved the efficiency of ELISA and Western blot analysis [32].

Enzyme-labelled antibody is the key to the ELISA signal output. The process of labelling involves the formation of a stable, covalent linkage between the enzyme and the antibodies mAb or pAb in which neither the antigen-combining site of the antibody nor the active site of the enzyme is functionally altered [33]. Various reporter enzymes, such as horseradish peroxidase (HRP), alkaline phosphatase (AP), or urease, can be attached to antibodies and proteins through the use of different coupling chemistries to ensure the maximum retention of activity of both enzyme and protein [34]. The enzyme converts the substrate, such as TMB, to a detectable product. If an ELISA has been developed properly, then the intensity of the signal produced when the substrate is added will be directly proportional to the amount of antigen captured in the plate and bound by the detection reagents. In the current study, the HRP-conjugated anti-human orthopneumovirus antibodies at 1000 and 1:10,000 of HRP-conjugated goat anti-rabbit IgG-Fc secondary antibody or HRP-conjugated goat anti-rat secondary antibody were selected as higher dilutions that could decrease the sensitivity for both antisera. This selected dilution is in agreement with previous studies where the working conjugate dilution was 1:100, 1:1000, and 1:10,000 [19,35,36].

The sensitivity of the ELISA was also highly influenced by the duration and the incubation temperature. Reactants should be incubated for the optimum time for a good antigen–antibody reaction to develop. A too short incubation period means that the antigen and the antibody may not have had sufficient time to form a strong bond. On the other hand, prolonged incubation periods may cause antigen–antibody complexes to dissociate [37]. In the current study, incubation at 37 °C for 1 h gave the best results.

Regarding the sensitivity and specificity, the current in-house diagnostic ELISA kit was determined in comparison to the rRT-PCR. The results obtained in the comparison between the indirect competitive inhibition ELISA and the rRT-PCR showed 93.3% sensitivity, because five samples out of 75 rRT-PCR positive samples were negative by ELISA. A possible explanation for the discrepancy in results for these five samples could be either analytical error or a decrease in virus particles in the clinical samples due to long-term storage. The specificity of the indirect competitive inhibition ELISA was 88.0%, because three samples out of 25 rRT-PCR negative samples had a positive test (Table 1); the discrepant results for the three samples can be attributed to the fact that the ELISA conjugate is a commercial HRP-conjugated goat anti-rat secondary antibody that may cross-react with IgA antibodies in NPA taken early in the infection. In the comparison of the indirect and direct antigen-capture ELISA with the rRT-PCR, the sensitivity was 90.6% and 86.6%, respectively.

Certain limitations in our assay development exist that need to be addressed. For the sensitivity test, the detection of low levels of the viral antigen can probably be improved by the evaluation of various critical parameters of ELISA and their optimization, and the cohort sample size was inadequate; therefore, future studies are needed on a large scale to determine the exact predictive value of these findings.

## 5. Conclusions

In conclusion, the findings of this study indicated that the two recombinant pPOE vectors (pPOE-F and pPOE-TF) were able to induce high titers of anti-human orthopneumovirus-specific antibodies. Validation of the ELISA method was performed in comparison to the rRT-PCR; the sensitivity hierarchy of these developed ELISA assays was as follows, from highest to lowest sensitivity: indirect competitive inhibition ELISA (93.3%) > indirect antigen-capture ELISA (90.6%) > direct antigen-capture ELISA (86.6%). The data presented here encourage the use of this kit in hospital settings as it is less expensive, more cost- and time-effective, and, therefore, suitable, and it could have extensive application prospects for the diagnosis of viral diseases. However, the detection of low levels of the viral antigen can probably be improved by the evaluation of various critical parameters of ELISA and their optimization. The development of the in-house diagnostic ELISA kits described in this study demonstrates that a specific, rapid, and sensitive test for human orthopneumovirus antigens could be successfully applied to clinical specimens.

## Figures and Tables

**Table 1:** Comparison of the results obtained with developed ELISA kits and PCR for human orthopneumovirus F protein detection.

	Positive	Negative	False Negative	False Positive	Total	Specificity	Sensitivity
rRT-PCR	75	25	0	0	100		
Direct antigen-capture ELISA	65	20	10	5	100	86.6%	80.0%
Indirect antigen-capture ELISA	68	21	7	4	100	90.6%	84.0%
Indirect competitive inhibition ELISA	70	22	5	3	100	93.3%	88.0%
